# iTRAQ Proteomic Analysis of Wheat (*Triticum aestivum* L.) Genotypes Differing in Waterlogging Tolerance

**DOI:** 10.3389/fpls.2022.890083

**Published:** 2022-04-25

**Authors:** Rui Yang, Murong Li, Matthew Tom Harrison, Shah Fahad, Mingmei Wei, Xiu Li, Lijun Yin, Aihua Sha, Meixue Zhou, Ke Liu, Xiaoyan Wang

**Affiliations:** ^1^Hubei Collaborative Innovation Center for Grain Industry, Yangtze University, Jingzhou, China; ^2^Agriculture College, Yangtze University, Jingzhou, China; ^3^Tasmanian Institute of Agriculture, University of Tasmania, Burnie, TAS, Australia; ^4^Hainan Key Laboratory for Sustainable Utilization of Tropical Bioresource, College of Tropical Crops, Hainan University, Haikou, China; ^5^Department of Agronomy, The University of Haripur, Haripur, Pakistan

**Keywords:** wheat, iTRAQ, proteomics, waterlogging, anthesis, abiotic stress, crop adaptation

## Abstract

Transient and chronic waterlogging constrains crop production in many regions of the world. Here, we invoke a novel iTRAQ-based proteomic strategy to elicit protein synthesis and regulation responses to waterlogging in tolerant (XM 55) and sensitive genotypes (YM 158). Of the 7,710 proteins identified, 16 were distinct between the two genotypes under waterlogging, partially defining a proteomic basis for waterlogging tolerance (and sensitivity). We found that 11 proteins were up-regulated and 5 proteins were down-regulated; the former included an Fe-S cluster assembly factor, heat shock cognate 70, GTP-binding protein SAR1A-like and CBS domain-containing protein. Down-regulated proteins contained photosystem II reaction center protein H, carotenoid 9, 10 (9′, 10′)-cleavage dioxygenase-like, psbP-like protein 1 and mitochondrial ATPase inhibitor. We showed that nine proteins responded to waterlogging with non-cultivar specificity: these included 3-isopropylmalate dehydratase large subunit, solanesyl-diphosphate synthase 2, DEAD-box ATP-dependent RNA helicase 3, and 3 predicted or uncharacterized proteins. Sixteen of the 28 selected proteins showed consistent expression patterns between mRNA and protein levels. We conclude that waterlogging stress may redirect protein synthesis, reduce chlorophyll synthesis and enzyme abundance involved in photorespiration, thus influencing synthesis of other metabolic enzymes. Collectively, these factors accelerate the accumulation of harmful metabolites in leaves in waterlogging-susceptible genotypes. The differentially expressed proteins enumerated here could be used as biological markers for enhancing waterlogging tolerance as part of future crop breeding programs.

## Introduction

Crop waterlogging (WL) may be caused by intense rainfall events, excessive irrigation, flash flooding, lateral surface or subsurface flow, and/or poor soil drainage (Herzog et al., 2016; Chang-Fung-Martel et al., 2017; Kaur et al., 2020). Waterlogging can hinder or cease plant growth by reducing oxygen availability in soil pore spaces in the root zone (Liu et al., 2020b). Anoxic soils and severe hypoxia then inhibit several physiological processes, including root water absorption, plant hormone relations, ion uptake, and transport and superoxide dismutase activities (Ghobadi et al., 2017; Manik et al., 2019). As well, waterlogging causes cascading changes in soil physico-chemical properties that can increase soil elemental toxicities, cause excessive loss of mineral nitrogen via leaching or greenhouse gas emissions (Harrison et al., 2016; Christie et al., 2020), and/or elevate concentrations of phenolics and volatile fatty acids within the plant (Ghobadi et al., 2017; Manik et al., 2019).

Plants can adapt to WL with changes in morphology, anatomy, and metabolism (Nguyen et al., 2018). Development of a shallow root system and formation of aerenchymatous adventitious roots are the main morphological/anatomical changes (Yamauchi et al., 2018; Ouyang et al., 2020), and are controlled by plant hormones such as ethylene, auxin, abscisic acid (ABA), cytokinin, jasmonates (JAs), and gibberellin (GA) (Nguyen et al., 2018). In rice, lysigenous aerenchyma and a barrier to radial O_2_ loss form in roots to mitigate WL stress by supplying O_2_ to the root tip (Nishiuchi et al., 2012).

In the past years, great efforts have been made to investigate the mechanism of WL tolerance at the molecular level. Many genes have been demonstrated to mediate WL in cotton (Zhu et al., 2022), rapes (Liu et al., 2021), maize (Du et al., 2017), cucumber (Kêska et al., 2021). Previous studies showed that 52 and 146 proteins were differentially expressed in tomato leaves and cucumber adventitious roots in response to WL stress, respectively (Ahsana et al., 2007; Xu et al., 2016). It has been demonstrated that 100 proteins were responsive to WL stress in different tissues of WL-sensitive and WL-tolerant barleys (Luan et al., 2018). Over-expression of the Kiwifruit AdPDC1 (Actinidia deliciosa pyruvate decarboxylase 1) could enhance WL resistance in transgenic *Arabidopsis thaliana* (Zhang et al., 2016).

Wheat (*Triticum aestivum* L.) is one of the most economically important cereal crops in the world. WL has reduced wheat grain yields by about 20–50% in the United Kingdom, North America, and Australia (Li et al., 2011). Some attempts have been made to investigate the regulation mechanism responding to WL in wheat. Transcripts of phenylalanine ammonia-lyase 6, cinnamoyl-CoA reductase 2, ferulate 5-hydroxylase 2 are involved in lignin biosynthesis, and have been shown to be repressed by WL (Nguyen et al., 2016). Genes regulating metabolism of hormones change under WL, which include *ACS7* and *ACO2* for ethylene biosynthesis, *TDC*, *YUC1*, and *PIN9* for indole acetic acid (IAA) biosynthesis/transport, *LOX8, AOS1, AOC1*, and *JAR1* for JA metabolism, *GA3ox2* and *GA2ox8* for GA metabolism, *IPT5-2, LOG1, CKX5*, and *ZOG2* for cytokinin metabolism, *NCED1* and *NCED2* for ABA biosynthesis (Nguyen et al., 2018). Anoxia under WL reduces the abundance of denitrification gene nirS in the rhizosphere of wheat (Hamonts et al., 2013). However, understanding of the molecular basis of WL tolerance is still limited in wheat.

WL has become a major constraint for wheat production in southeast of China due to excessive rainfall during the growing season, which is especially severe during the critical grain formation periods of anthesis and maturation (Liu et al., 2020b; Yan et al., 2022). Proteomics is a useful and important method for investigating crop responses to stress by detecting changes in expression and post-translational modification of proteins (Komatsu et al., 2012). Proteomic techniques have been performed to investigate proteins in response to WL in cotton (Gong et al., 2017), soybean (Alam et al., 2010), cucumber (Xu et al., 2016), barley (Luan et al., 2018), etc. Proteomic approaches have also been successfully used to perform proteomic profiles in response to flooding, drought, high temperature, salt, metal stresses in wheat (Komatsu et al., 2014). In this study, our primary aim was to define the key differences in proteomic and transcriptional levels under waterlogging stress conditions of tolerant and susceptible wheat genotypes. This knowledge would be expected to provide important insights into physiological and molecular mechanisms associated with waterlogging tolerance in wheat leaves, enabling future crop breeders to better develop waterlogging tolerant genotypes.

## Materials and Methods

### Plant Growth Conditions and Treatments

The wheat cultivars Xiangmai 55 and Yangmai 158 were used in the screen for WL-responsive proteins. They were sown in the farm of Yangtze University located in Jingzhou, Hubei Province, China in growing season on November 15, 2017. The soil type in the uppermost 20 cm was a clay loam having the following biological and nutritive qualities: Organic matter content (10.5 g⋅kg^–1^), available N (33.41 mg⋅kg^–1^), available P (45.37 mg⋅kg^–1^) and available K (80.26 mg⋅kg^–1^).

Field experiments were arranged in a split-plot design with treatments as the main plots and cultivars as the subplots. Treatments included a waterlogging treatment that initiated at flowering stage for 15 days, and a non-waterlogged control. Except for waterlogged treatments, groundwater was deep (more than 2 m from the surface). Three replicates were performed per treatment for each variety, and the plot areas were 12 m^2^ (2 m × 6 m). At the sowing stage, the base application rate was 90 kg/hectare of pure nitrogen from the application of compound fertilizer, and the ratio of available nitrogen N, phosphorus P_2_O_5_ and potassium K_2_O in compound fertilizer was 26:10:15. At the jointing stage, pure nitrogen was applied at 90 kg/hectare in the form of urea. At the trefoil stage, 224 plants m^–2^ remained. Otherwise, regular field management practices were employed. Wheat plants for both cultivars at anthesis stage were subjected to control and waterlogging treatment for 7 days. When 50% plants begin to bloom, in which the plants height are more than 80 cm, the plots were submerged in 2 cm- depth water as waterlogging treatment. A total of 10 plants was selected per group, and three biological replicates were conducted for each treatment. The flag leaves were collected, immediately frozen, and stored in liquid nitrogen for protein and RNA extraction for qRT-PCR.

### Protein Extraction, Digestion, and iTRAQ Labeling

Total protein was extracted using the cold acetone method. Samples were ground in liquid nitrogen and dissolved in 2 mL lysis buffer (8 M urea, 2% SDS, 1x Protease Inhibitor Cocktail (Roche Ltd. Basel, Switzerland). Subsequently, sonication on ice for 30 min and centrifugation at 13,000 rpm for 30 min at 4°C were conducted. Proteins were precipitated with ice-cold acetone at –20°C, and the precipitate was cleaned with acetone three times and re-dissolved. The protein quality was determined by SDS-PAGE (Supplementary Figure 3).

Bicinchoninic acid assay (BCA; Pierce, MA, United States) was used to determine the protein concentration. The 100-μg protein from the previous step was transferred into a new tube and adjusted to a final concentration of 1 μg/μL, and then treated with 11 μL of 1M DTT (DL-Dithiothreitol) at 37°C for 1 h. Then we used 120 μL of the 55 mM iodoacetamide and incubated the mixture for 20 min at room temperature in the dark.

For each sample, proteins were precipitated with ice-cold acetone, then re-dissolved in 100 μL TEAB (0.25M, pH8.5). Then samples were tryptic digested with trypsin (Promega, Madison, WI) at 37°C for 4 h (trypsin: protein 1:100). The resultant peptide mixture was labeled with iTRAQ tags 113 through 118. The labeled samples were combined and dried in vacuum.

### Strong Cation Exchange Fractionation and Liquid Chromatography–Tandem Mass Spectrometry Analysis

The combined labeled samples were bound to a strong cation exchange (SCX) fractionation column connected with a high performance liquid chromatography (HPLC) system. The peptide mixture was re-dissolved in the buffer A (20 mM ammonium formate in water, pH10.0), and then fractionated by high pH separation using Ultimate 3000 system (Thermo Fisher Scientific, MA, United States) connected to a reverse phase column (Gemini-NX 3u C18 110A column, 2.0 mm × 150 mm, 3 μm, (Waters Corporation, MA, United States). High pH separation was performed using a linear gradient starting from 5 to 45% buffer B (20 mM ammonium formate in 80% ACN, pH 10.0) in 40 min. The column flow rate was maintained at 0.2 mL/min and column temperature was maintained at 30°C. A total of 12 fractions were collected, and each fraction was dried in a vacuum concentrator for the next step.

Peptide fractions were resuspended with 30 μL solvent C (water with 0.1% formic acid), respectively, and separated by nanoLC and analyzed by electrospray tandem mass spectrometry. The experiments were performed on an Easy-nLC 1000 system (Thermo Fisher Scientific, MA, United States). A total of 10 μL peptide sample was loaded onto the trap column (Thermo Fisher Scientific Acclaim PepMap C18, 100 μm × 2 cm), with a flow of 10 μL/min for 3 min and subsequently separated on the analytical column (Acclaim PepMap C18, 75 μm × 15 cm) with a linear gradient, from 3 to 32% solvent D (ACN with 0.1% formic acid) in 120 min. The column flow rate was maintained at 300 nL/min.

The fusion mass spectrometer was run in the data-dependent mode to switch automatically between MS and MS/MS acquisition. Survey full-scan MS spectra (m/z 350–1,550) were acquired with a mass resolution of 120 K, followed by sequential high energy collisional dissociation MS/MS scans with a resolution of 30 K. The isolation window was set as 1.6 Da. MS/MS fixed first mass was set at 110. In all cases, one microscan was recorded using dynamic exclusion of 45 s.

### Database Search and Quantification

The mass spectrometry data were transformed into MGF (Mascot generic format) files with Proteome Discovery 1.2 (Thermo Fisher Scientific, PA, United States) and analyzed using Mascot software version 2.3.2 (Matrix Science, London, United Kingdom). Mascot database was set up for protein identification using Triticum aestivum L database in NCBI nr (release 2017_03); SwissProt/UniprotKB (release 2018_06) and International Protein Index (IPI; version 3.16). Trypsin/P was chosen as the enzyme with two missed cleavages allowed; Peptide tolerance was set at 10 ppm, and Mascot was searched with a fragment ion mass tolerance of 0.050 Da; a parent ion tolerance of 10.0 PPM. Significance threshold *p* < 0.05 (with 95% confidence). The average values of the biological replicates were used to indicate the final protein abundances for each sample. Proteins with a 1.2-fold change between samples and a *p*-value less than 0.05 were determined as differentially expressed proteins (DEPs).

### Gene Ontology and Kyoto Encyclopedia of Genes and Genomes Enrichment Analysis

The DEPs were selected for functional enrichment analysis. The hypergeometric test was used to determine significant enrichment of GO terms relative to the background. The *p*-value was adjusted with FDR Correction, setting FDR ≤ 0.05 as a threshold. The GO terms with FDR ≤ 0.05 were defined as significantly enriched GO terms. Likewise, Kyoto encyclopedia of genes and genomes (KEGG) pathway enrichment was also performed with KEGG database (Pan et al., 2016). The calculated *p*-value was adjusted with FDR Correction, setting FDR ≤ 0.05 as a threshold.

### RNA Extraction and Quantitative Real-Time PCR

Total RNA was extracted using the TRIZOL reagent (Invitrogen, Carlsbad, CA, United States). Then RNA samples were reverse-transcribed using the RevertAid™ First Strand cDNA Synthesis Kit (Thermo Fisher Scientific, MA, United States) according to the manufacturer’s protocol. Each reaction was conducted in 10 μL mixture containing 5 μL of SYBR green [SYBR^@^ Premix Ex Taq™ (TliRNaseH Plus), TAKARA, Japan], 0.6 μL forward and reverse primers (10 μM), 2 μL cDNA template, and 2.4 μL ddH_2_O. The Quantitative Real-Time PCR (qRT-PCR) reactions were performed with CFX96™ Real-Time PCR Detection System (Bio-Rad, United States). The primers used for qPCR are listed in Supplementary Table 6. The reactions for each gene were conducted in triplicate with the thermal cycling conditions as follows: 95°C for 30 s, followed by 40 cycles of 95°C for 5 s and 57°C for 30 s. The primer specificity was confirmed by melting curve analysis. Relative expression levels of the genes were calculated using the 2^–ΔΔCT^ method (Kim et al., 2013).

## Results

### Phenotypic and Physiological Analysis of Two Varieties

**FIGURE 1:**
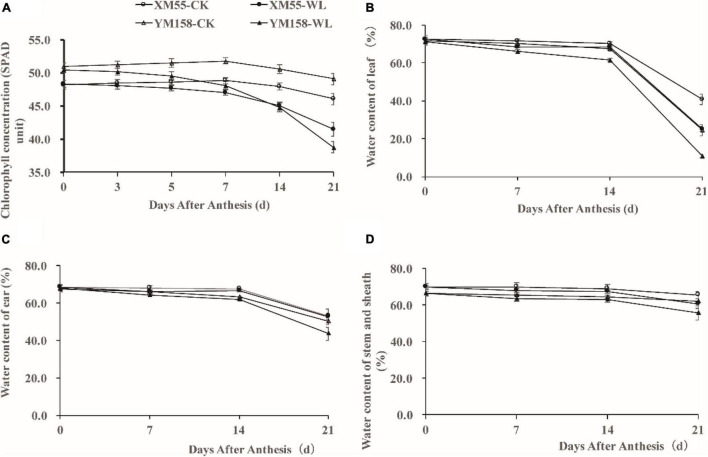
Phenotypes of XM 55 and YM 158 under waterlogging and control conditions. **(A)** Dynamic changes of chlorophyll concentration (SPAD unit) of the last expanded leaf after 7 days waterlogging at anthesis. **(B)** Dynamic changes of water content of leaf after waterlogging at anthesis between different varieties. **(C)** Dynamic changes of ear water content after waterlogging at anthesis between different varieties. **(D)** Dynamic changes of water content of stem and sheath after waterlogging at anthesis between different varieties. Vertical bars indicate standard error of the mean (*n* = 3).

WL is known to induce chlorosis and early senescence of leaves (Romina et al., 2014); Firstly, we detected the chlorophyll concentration in expanded flag leaves of WL-tolerant variety XM 55 and WL-sensitive variety YM 158 by measuring SPAD (soil-plant analysis development) at the anthesis stage. The SPAD value of XM 55 was higher than that of YM 158 during 0–7 days, and it was less than or equal to that of YM 158 during 7–21 days under WL (Figure 1A). However, the SPAD value of XM 55 was higher than that of YM 158 between 0 and 21 days under normal conditions (CK) (Figure 1A). The SPAD value of XM 55 under normal conditions decreased below that of WL treated XM 55 after 7 days, whereas it was decreased below that of YM 158 under CK at 5 days (Figure 1A). The reductions of SPAD in XM 55 from 0 to 7 days, 7–14 days, and 14–21 days under WL were 2.7, 4.2, and 7.8%, whereas they were 4.7, 6.9, and 13.4% in YM 158, respectively.

Soil WL causes serious hypoxia in plant roots, obstructs root growth and development, decreases root activity, and decreases root water permeability; this affects plant water uptake and transpiration rate, thereby leading to water deficit in plants and alterations in the above-ground distribution of water (Lina et al., 2012; Romina et al., 2014). We also measured the above-ground water contents in the two varieties. Under WL, the water contents in flag leaves, ears, and stem and sheath were significantly higher in XM 55 than in YM 158 from 7 to 21 days, whereas this pattern occurred from 14 to 21 days under CK (Figures 1B–D).

WL at elongation or post-anthesis is known to affect grain yield, as well as accumulation and remobilization of dry matter in wheat (Lina et al., 2012). We measured the changes of aboveground dry matter accumulation (DMA), yield, and yield-related traits of the two varieties. WL had different effects on XM 55 and YM 158. The DMA at anthesis (DMA1) before WL were roughly similar between XM 55 and YM 158, but under WL, DMA values were decreased by 12.5 and 20.5% in XM 55 and YM 158 relative to the CK control at the mature stage, respectively (Table 1). At the same time, kernels per spike, 1,000-kernel weight, grain yield weight, and harvest index were decreased under WL by 3.3, 18.1, 26.2, and 15.9% in XM 55, and by 10.8, 36.2, 36.8, and 21.8% in YM 158 relative to their CK control values, respectively (Table 1). Clearly, WL had greater effects on YM 158 instead of XM 55, especially the 1,000-kernel weight and grain yield weight. Overall, it could be inferred that XM 55 showed better WL tolerant than YM 158.

**TABLE 1:** Effect of waterlogging on yield and yield components of wheat.

Cultivar	Treatment	DMA1 (g stem^–1^)	DMA2 (g stem^–1^)	Kernel per spike	1,000-kernel weight (g)	Grain yield weight (g stem^–1^)	Harvest index
XM 55	CK	2.07a	3.21b	43.31b	29.33b	1.41b	0.44b
	WL	2.1a	2.81c	41.87b	24.02c	1.04c	0.37c
	(WL-CK)/CK	/	0.125	0.033	0.181	0.262	0.159
YM 158	CK	2.19a	3.57a	45.47a	34.47a	1.63a	0.46a
	WL	2.17a	2.84c	41.56b	25.31c	1.03c	0.36c
	(WL-CK)/CK	/	0.205	0.108	0.362	0.368	0.218

*The lowercase letters indicate significant differences at P < 0.05 among treatments as determined by Duncan’s Multiple Range Test. DMA1, aboveground dry matter accumulation at anthesis before waterlogging; DMA2, aboveground dry matter accumulation at maturity; CK, Control; WL, Waterlogging.*

### Waterlogging Induced Proteome Change in XM 55 and YM 158

**FIGURE 2:**
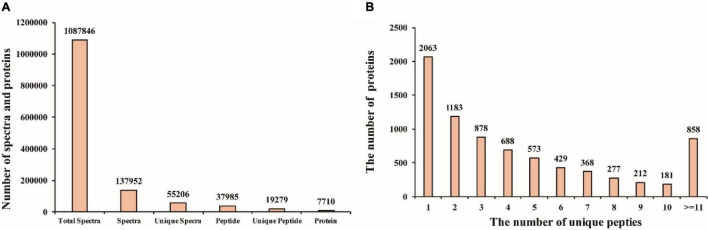
Mass spectrometry analysis and protein identification. **(A)** The number of spectra and proteins. **(B)** The number of proteins with different unique peptides.

To further explore the molecular mechanisms that mediate different responses to WL, iTRAQ method was used to analyze proteome changes in flag leaf of both cultivars. After protein extraction, enzyme digestion, iTRAQ labeling, equal mixing and SCX pre-separation, all samples were subjected to liquid chromatography–tandem mass spectrometry (LC-MS/MS) in three independent replicates. In the present study, a total of 1,087,846 spectra were detected, among which, 37,952 could be matched and 55,206 were unique spectra, and 37,985 peptides could be identified with 19,279 being unique peptides, and 7,710 proteins were identified (Figure 2A); the proteins identified in the flag leaf of the XM 55 and YM 158 plants were supported by unique peptides. Of those proteins, 54.0% (4,164) were inferred from more than three unique peptides (Figure 2B).

### Pairwise Comparison of Protein Profiling in XM 55 and YM 158 Under Waterlogging

**FIGURE 3:**
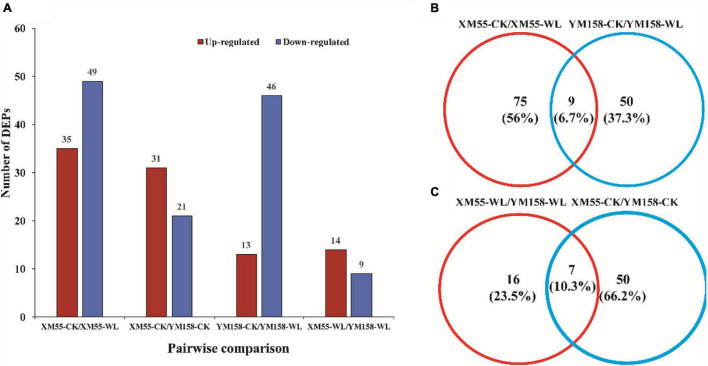
Quantitative and Venn analysis of the proteome of two wheat cultivars under different treatments. **(A)** Quantitative analysis of the proteome between the waterlogging treated and control samples. **(B)** Venn analysis of two wheat cultivars under different treatments. **(C)** Venn analysis of different treatments in different wheat cultivars; XM 55 and YM 158 are the two cultivars; CK, control; WL, waterlogging.

To identify differentially expressed proteins (DEPs) between the two cultivars in response to WL, proteins with more than a 1.2-fold change in abundance (*p* < 0.05) between XM 55 and YM 158 under WL and CK were investigated. Based on this criterion, 23 DEPs (14 up-regulated and 9 down-regulated) showed differential expressed between XM 55 and YM 158 under WL (XM 55-WL/YM 158-WL), and 52 DEPs (31 up-regulated and 21 down-regulated) were differently expressed between XM 55 and YM 158 under CK (XM 55-CK/YM 158-CK) (Figure 3). At the same time, 7 proteins (i.e., TRIAE_CS42_2BL_TGACv1_130584_AA0414140.1, TRIAE_CS 42_2BL_TGACv1_131439_AA0427700.2, TRIAE_CS42_4BL_ TGACv1_321826_AA1065960.1, TRIAE_CS42_2BL_TGACv1 _132610_AA0438610.1, TRIAE_CS42_6BL_TGACv1_503168_A A1627380.1, TRIAE_CS42_6BL_TGACv1_503168_AA162738 0.2, TRIAE_CS42_6BL_TGACv1_503168_AA1627380.3) were differentially accumulated in both (XM 55-WL/YM 158-WL) and (XM 55-CK/YM 158-CK), which might indicate cultivar specific protein accumulation irrespective of WL treatment (Figure 3, Table 2, and Supplementary Table 1). Excluding these 7 overlapping DEPs, a total of 16 DEPs were remained between XM 55 and YM 158 under WL (Figure 3B); of these, 11 DEPs were up-regulated, including members of Fe-S cluster assembly factor, heat shock cognate 70 kDa protein, GTP-binding protein SAR1A-like, and CBS domain-containing protein, respectively. The 5 down-regulated proteins were photosystem II reaction center protein H, carotenoid 9, 10 (9′, 10′)-cleavage dioxygenase-like, psbP-like protein 1, and mitochondrial ATPase inhibitor (Table 2).

**TABLE 2:** Differentially expressed proteins between XM 55 and YM 158 under WL.

Gene ID	Log2_FC (XM55/YM158)	Protein description	Functional category
**UP-regulated**			
TRIAE_CS42_2BL_TGACv1_130584_AA0414140.1	1.63	Ubiquinol oxidase 4	Redox
TRIAE_CS42_2BL_TGACv1_131439_AA0427700.2	1.50	Superoxide dismutase [Mn]	Redox
TRIAE_CS42_4BL_TGACv1_321826_AA1065960.1	1.18	Heat shock protein 101	Stress response
TRIAE_CS42_3AL_TGACv1_195570_AA0651350.1	0.33	Fe-S cluster assembly factor HCF101	Chloroplast
TRIAE_CS42_3AL_TGACv1_195570_AA0651350.2	0.33	Fe-S cluster assembly factor HCF101	Chloroplast
TRIAE_CS42_3AL_TGACv1_195570_AA0651350.3	0.33	Fe-S cluster assembly factor HCF101	Chloroplast
TRIAE_CS42_3DL_TGACv1_250912_AA0874940.1	0.33	Fe-S cluster assembly factor HCF101	Chloroplast
TRIAE_CS42_3DL_TGACv1_250912_AA0874940.2	0.33	Fe-S cluster assembly factor HCF101	Chloroplast
TRIAE_CS42_2BL_TGACv1_131039_AA0421600.2	0.32	Heat shock cognate 70 kDa protein 2-like	Transcription
TRIAE_CS42_6DL_TGACv1_526647_AA1688990.1	0.32	Heat shock cognate 70 kDa protein 2-like	Transcription
TRIAE_CS42_3AS_TGACv1_211332_AA0688720.1	0.31	GTP-binding protein SAR1A-like	GTP binding
TRIAE_CS42_3DS_TGACv1_272355_AA0919480.1	0.31	GTP-binding protein SAR1A-like	GTP binding
TRIAE_CS42_6DL_TGACv1_526455_AA1684150.2	0.29	CBS domain-containing protein	
TRIAE_CS42_6DL_TGACv1_526455_AA1684150.3	0.29	CBS domain-containing protein	
**Down-regulated**			
AIG90456	–0.26	Photosystem II reaction center protein H	Plastid
TRIAE_CS42_5DS_TGACv1_456540_AA1473460.1	–0.36	Carotenoid 9, 10 (9′, 10′)-cleavage dioxygenase-like	Stress response
TRIAE_CS42_4BS_TGACv1_330468_AA1107820.2	–0.67	psbP-like protein 1, chloroplastic	Metabolic
TRIAE_CS42_4BS_TGACv1_329474_AA1101780.1	–0.87	Mitochondrial ATPase inhibitor	Photorespiration
TRIAE_CS42_4BS_TGACv1_329474_AA1101780.3	–0.87	Mitochondrial ATPase inhibitor	Photorespiration
TRIAE_CS42_2BL_TGACv1_132610_AA0438610.1	–1.29	Aminomethyltransferase	Redox
TRIAE_CS42_6BL_TGACv1_503168_AA1627380.1	–0.78	Uncharacterized protein	
TRIAE_CS42_6BL_TGACv1_503168_AA1627380.2	–0.78	Uncharacterized protein	
TRIAE_CS42_6BL_TGACv1_503168_AA1627380.3	–0.78	Uncharacterized protein	

### Proteomic Dynamics in XM 55 and YM 158 Between Waterlogging and Normal Conditions

The DEPs in XM 55 and YM 158 between WL and CK were identified. There were 84 DEPs (35 up-regulated and 49 down-regulated) between XM 55-WL and XM 55-CK, and 59 DEPs (13 up-regulated and 46 down-regulated) between YM 158-WL and YM 158-CK (Figure 3 and Supplementary Tables 2, 3). Most proteins responsive to WL were specific to XM 55 or YM 158. However, 9 proteins were differentially expressed in both XM 55-WL/XM-CK and YM 158-WL/YM 158-CK (Figure 3 and Supplementary Tables 2, 3), which might be WL responsible proteins with non-cultivar specificity. These proteins were 3-isopropylmalate dehydratase large subunit, solanesyl-diphosphate synthase 2, DEAD-box ATP-dependent RNA helicase 3, and three predicted or Uncharacterized proteins. 3-isopropylmalate dehydratase catalyzes, the stereo-specific isomerization of 2-isopropylmalate and 3-isopropylmalate participate in the biosynthesis of leucine.

### Functional Categorization, Gene Ontology, and Kyoto Encyclopedia of Genes and Genomes Pathway Enrichment Analysis of the Differentially Expressed Proteins

**FIGURE 4:**
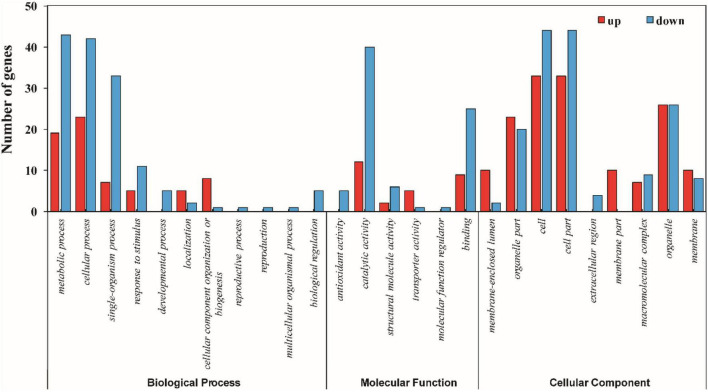
GO annotation of differentially expressed proteins between XM 55 and YM 158 under WL.

The functional information of all differentially accumulated proteins in Figure 3 were obtained by searching against the UniProt-GOA database, which were assigned to three categories based on GO annotation, that is, cellular compartment, biological process, and molecular function. The differentially expressed proteins among XM 55 and YM 158 under WL belonged to eight biological processes, 11 cellular compartments, and two different molecular functions (Figure 4 and Supplementary Table 4). In terms of biological processes, metabolic process, cellular process, and cellular component organization or biogenesis were the three major groups. It was suggested that the DEPs may be involved in primary metabolic processes, and these impart differential WL tolerances to XM 55 and YM 158. Cell, cell part, and membrane-enclosed lumen were the top three cellular compartments, implying that various changes in cell structure had effects on tolerance to WL among different varieties. Binding was the major molecular functional groups, and a small amount of differentially accumulated proteins were involved in catalytic activity, which showed that protein binding affects tolerance to WL.

The differentially expressed proteins among XM 55 or YM 158 under WL and CK belonged to 11 or 8 biological processes, 9 or 11 cellular compartments, and 6 or 3 molecular functions (Supplementary Figures 1, 2 and Supplementary Table 4), respectively. Metabolic process, cellular process, and single-organism process were both the three major biological processes. Cell, cell part, and organelle were both the top three cellular compartments. Catalytic activity and binding were both the two-major molecular functional groups. Those results indicated that primary metabolic processes, cell structure, and catalytic activity were generally affected by WL regardless of cultivar tolerance.

To characterize the functional consequences of the differentially expressed proteins associated with WL, the enriched pathways were assigned based on KEGG terms. The results indicated that the proteins related to terpenoid backbone biosynthesis, amino sugar and nucleotide sugar metabolism, and fructose and mannose metabolism were affected by WL in XM 55, whereas terpenoid backbone biosynthesis and fatty acid biosynthesis were affected in YM 158. Tuberculosis and RNA degradation were affected by WL both in XM 55 and YM 158 (Supplementary Table 5).

### Correlation of Differentially Accumulated Protein With mRNA Expression

**FIGURE 5:**
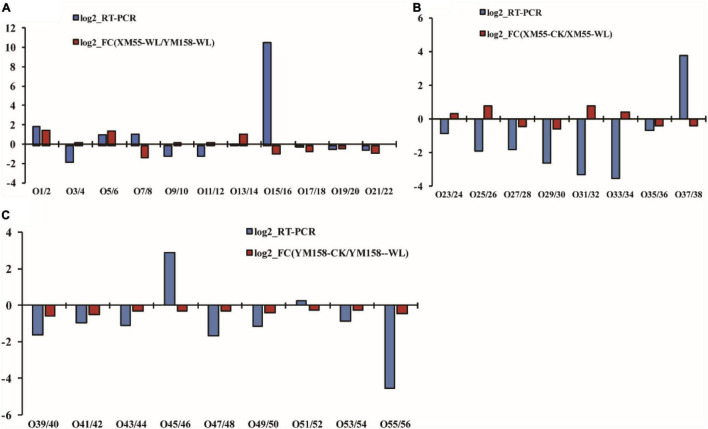
Correlation of differentially expressed protein at transcript and translation level. Differences in protein expression and qRT-PCR between XM 55 and YM 158 under waterlogging stress. **(A)** Differences in protein expression measured by iTRAQ and quantitative real-time reverse transcription-PCR (qRT-PCR) in XM 55 **(B)** and YM 158 **(C)** under WL and CK. Log2-RT-PCR represents RNA expression level; Log2-FC represents the differences in protein expression level.

To verify the correlation between the expression levels of the differentially expressed proteins and their mRNAs, the mRNA expression levels of 28 differentially expressed proteins were analyzed using qRT-PCR method (Supplementary Table 6). Among them, 16 genes exhibited consistent expression patterns with their proteins, whereas 12 showed discrepancies between protein accumulation and mRNA expression (Figure 5). The discrepancy between protein accumulation and mRNA expressions might be ascribed to translational and posttranslational regulatory processes or feedback loops between the processes of mRNA translation and protein degradation (Ahsana et al., 2007). These results were consistent with previous studies that transcription patterns do not always directly correlate with protein expression levels (Yan et al., 2006; Luan et al., 2018).

## Discussion

Crop growth and development is inhibited by waterlogging stress, with anthesis being the most sensitive stage (Liu et al., 2020a,b; Yan et al., 2022). In this study, we compared the waterlogging tolerance of two wheat genotypes, and found that waterlogging impacted the chlorophyll content, water content, grain weight and its components, and accumulation of dry matter after anthesis in both varieties at anthesis. Notably, the degree of waterlogging influence varied between different wheat varieties, that could explain why XM 55 was less sensitive to water stress than YM 158 in this study. Such results have synergies with effects of defoliation and nitrogen stress on chlorophyll content, suggesting plant response and recovery to some abiotic and nutritive stresses may be similar (Harrison et al., 2011, 2012a,b; Alcock et al., 2015; Christie et al., 2018).

We invoked iTRAQ and HPLC-MS techniques to analyze flag leaf protein expression patterns to determine the effects of waterlogging stress in two genotypes (i.e., XM 55 and YM 158). Overall, the number of DEPs identified in the pairwise comparison was relatively lower than the previous studies (Jia et al., 2009; Kim et al., 2013), inferring that the different tissues (e.g., leaf, root, stem) and number of biological replicates could determine the number of DEPs to some extent. Meanwhile, the treatment and sampling stage in the experiment design also could decrease the difference of genetic and proteomic expression. On the other hand, the low number of DEPs identified in the comparisons contributed to finding the key proteins and pathways that play roles in WL tolerance of wheat. A total of 11 up-regulated proteins in XM 55 were identified in response to WL that were involved in iron acquisition, proteins folding assistant, cargo secretion, abiotic stresses, whereas 5 proteins were down-regulated, which participated in light energy usage, strigolactone biosynthesis, vesicle-mediated secretion. The differential tolerance of waterlogging between XM 55 and YM 158 might be ascribed to those differentially accumulated proteins. In detail, the DEPs related to Fe/S clusters participate in diverse cellular processes in almost all organisms, which include respiration, metabolism, DNA replication and repair, and regulation of gene expression (Beinert et al., 1997; Lill, 2009). The gene *sufT*, which is involved in the Fe/S cluster assembly pathway, has been reported that it is necessary for effective symbiosis to enhance iron availability (Sasaki et al., 2016). Heat shock cognate 70 kDa protein is a chaperone that assists in the folding of other proteins *in vivo*; this protein increased expression in sugarcane plants subjected to WL (Khan et al., 2014). Over expression of CBS domain-containing protein could enhance tolerance to different abiotic stresses in maize (Dong et al., 2020) and Arabidopsis (Hao et al., 2016). These proteins were up-regulated in XM 55 compared to YM 158 under WL, indicating that their enhanced accumulation may be responsible for WL tolerance. Interestingly, some potential critical proteins which have been considered as critical factors for WL tolerance in wheat were not differentially expressed in this study. For instance, Wang et al. (2016) revealed that S-adenosylmethionine synthtase (SAMS), involved in ethylene biosynthesis pathway, was upregulated by WL stress in wheat. As well, alcohol dehydrogenases involved in carbohydrate metabolism were upregulated under WL stress (Kaur et al., 2021; Lee et al., 2021). Notably, these key candidates were not observed in this study. This suggests that alcohol dehydrogenases are tissue-specific proteins, and perhaps also that they are only expressed in root tissues, rather than the flag leaves of wheat.

Photosystem II (PSII) reaction center protein H and psbP are constituents of PS II, which uses light energy to split water into chemical products (Vinyard et al., 2013). Carotenoid cleavage dioxygenases (CCDs) cleave carotenes and xanthophylls to apocarotenoids, which may mediate strigolactone biosynthesis and are responsive to phosphorus deficiency (Pan et al., 2016), wounding, heat, and osmotic stress (Rubio-Moraga et al., 2014). The ATPases play roles in diverse cellular activities such as vesicle-mediated secretion, membrane fusion, cellular organelle biogenesis, and hypersensitive responses (HR) in plants (Baek et al., 2011). These proteins were down-regulated in XM 55 compared to YM 158 under WL, suggesting that WL tolerance might be associated with reduced energy production, changes of hormone content and cellular activities in plants.

In addition, 9 DEPs were detected in both WL tolerant and non-tolerant varieties (Figure 3 and Supplementary Tables 2, 3), which were involved in leucine biosynthesis, plastoquinone biosynthesis, and ribosomal structure remodeling, indicating they played basic roles in tolerance of WL stress. GO and KEGG pathway analysis indicated that proteins involving in primary metabolic processes, cell structure, protein binding determined the different tolerance to WL between XM 55 and YM 158. Compared with the control group, the proteins upregulated in WL group also play important roles in tolerance of WL stress. For instance, Solanesyl-diphosphate synthase 2 is involved in plastoquinone biosynthesis, which regulates gene expression and enzyme activities as a photosynthetic electron carrier, and plays a central photoprotective role as an antioxidant (Ksas et al., 2015). DEAD-box ATP-dependent RNA helicase 3 is involved in ribosomal structure and it was shown to be markedly suppressed after salt treatment in cotton (Gong et al., 2017). These proteins were responsive to waterlogging without cultivar specificity, indicating that the leucine, reactive oxygen species, and the ribosome may play roles in basic defense to WL.

qRT-PCR analysis indicated that consistent expression patterns were observed between mRNAs and proteins for most selected proteins. However, a discrepancy was also identified for several proteins between protein accumulation and mRNA expression. It could be suggested that transcription patterns do not always directly correlate with protein expression levels (Yan et al., 2006; Luan et al., 2018), which might be ascribed to translational and posttranslational regulatory processes or feedback loops between the processes of mRNA translation and protein degradation (Ahsana et al., 2007).

## Conclusion

We have shown that many proteins were differentially expressed under transient waterlogging of wheat. We conclude that waterlogging stress may redirect protein synthesis to reduce chlorophyll synthesis and the concentration of enzymes involved in photorespiration, thus influencing the synthesis of metabolic enzymes. As well, we suggest that reduced chlorophyll content may accelerate the accumulation of harmful metabolites in leaves. We suggest that differentially expressed proteins enumerated here could be used as biological markers for developing future waterlogging tolerant genotypes in crop breeding programs.

## Data Availability Statement

The data presented in the study are deposited in the Mendeley data repository, accession number DOI: 10.17632/xwrpkcmvjb.1.

## Author Contributions

XW designed the experiments. RY and ML conducted iTRAQ experiments. AS conducted biologic information analysis. XL conducted qRT-PCR. LY conducted waterlogging phenotype collection. MH, MZ, KL, and XW revised and edited the manuscript and also provided advice on the experiments. All authors contributed to the article and approved the submitted version.

## Conflict of Interest

The authors declare that the research was conducted in the absence of any commercial or financial relationships that could be construed as a potential conflict of interest.

## Publisher’s Note

All claims expressed in this article are solely those of the authors and do not necessarily represent those of their affiliated organizations, or those of the publisher, the editors and the reviewers. Any product that may be evaluated in this article, or claim that may be made by its manufacturer, is not guaranteed or endorsed by the publisher.

## Supplementary Material

Supplementary Figure 1 GO annotation of differentially expressed proteins between WL and CK in XM 55.

Supplementary Figure 2 GO annotation of differentially expressed proteins between WL and CK in YM 158.

Supplementary Figure 3 SDS-PAGE analysis for the samples.

## Abbreviations

qRT-PCR: quantitative real-time PCR
ABA: abscisic acid
JAs: Jasmonates
AdPDC1: Actinidia deliciosa pyruvate decarboxylase 1
IAA: Indole acetic acid
GA: Gibberellin
PSII: Photosystem II
HR: Hypersensitive responses
DEGs: different expression genes
WL: Waterlogging
CK: Normal conditions
DMA: Dry matter accumulation
CCDs: Carotenoid cleavage dioxygenases
GO: Gene Ontology
SCX: Strong cation exchange
LC-MS/MS: Liquid chromatography–tandem mass spectrometry.
