# Supplementary figures and images for: iTRAQ Proteomic Analysis of Wheat (Triticum aestivum L.) Genotypes Differing in Waterlogging Tolerance
Source: Front Plant Sci. 2022 Apr 25;13:890083. doi: 10.3389/fpls.2022.890083 (PMC9084233; doi:10.3389/fpls.2022.890083)

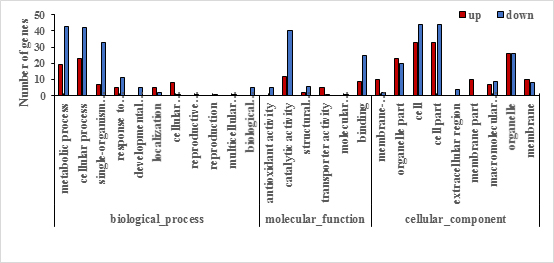

Supplement: Supplementary Figure 1 — GO annotation of differentially expressed proteins between WL and CK in XM 55. [file Image_1.JPEG]

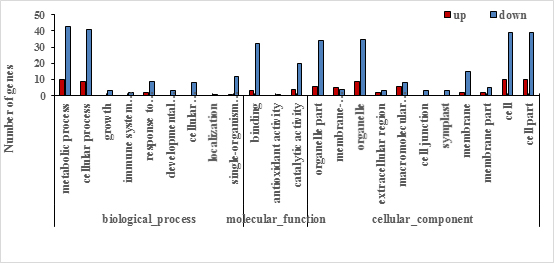

Supplement: Supplementary Figure 2 — GO annotation of differentially expressed proteins between WL and CK in YM 158. [file Image_2.JPEG]

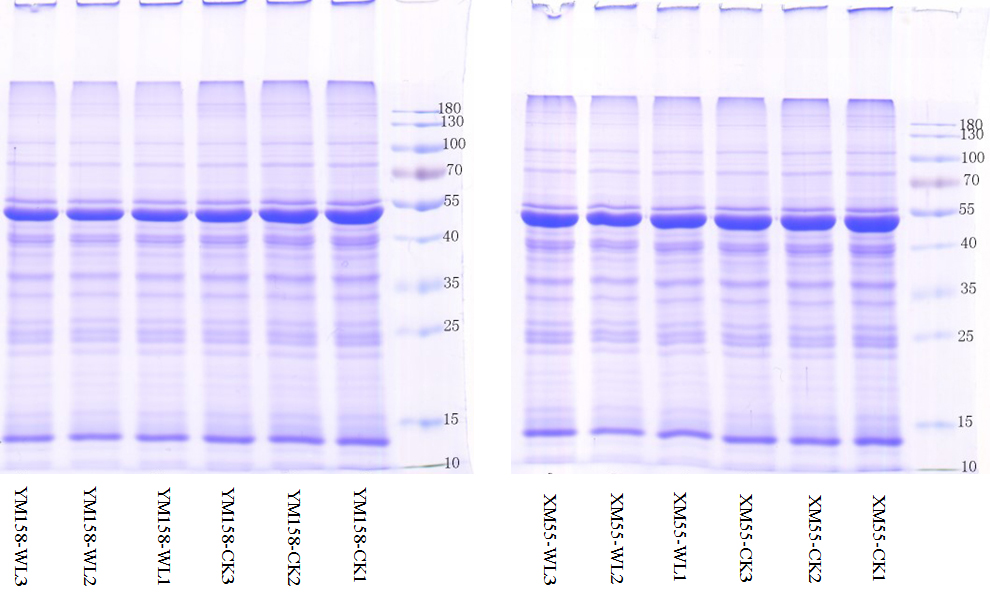

Supplement: Supplementary Figure 3 — SDS-PAGE analysis for the samples. [file Image_3.jpg]
