# Supplementary material for: iTRAQ Proteomic Analysis of Wheat (Triticum aestivum L.) Genotypes Differing in Waterlogging Tolerance
Source: Front Plant Sci. 2022 Apr 25;13:890083. doi: 10.3389/fpls.2022.890083 (PMC9084233; doi:10.3389/fpls.2022.890083)
Supplement: Supplementary file 4 [file Table_1.DOCX]

**TableS1. Differentially expressed proteins between XM55 and YM158 under control**

| Gene ID | log2_FC(XM/YM) | Protein Description | Functional Category |
| --- | --- | --- | --- |
| UP-regulated | | | |
| TRIAE_CS42_4BL_TGACv1_321826_AA1065960.1 | 1.332587442 | heat shock protein 101 | stress response |
| TRIAE_CS42_2BL_TGACv1_131439_AA0427700.2 | 1.258701985 | Superoxide dismutase [Mn], mitochondrial | redox |
| TRIAE_CS42_2BL_TGACv1_130584_AA0414140.1 | 0.83029111 | Ubiquinol oxidase 4, chloroplastic/chromoplastic | redox |
| TRIAE_CS42_6DL_TGACv1_527144_AA1699290.1 | 0.714082087 | [photosystem II 10 kDa polypeptide, chloroplastic](https://blast.ncbi.nlm.nih.gov/Blast.cgi) | stress response |
| TRIAE_CS42_6DL_TGACv1_527144_AA1699290.2 | 0.714082087 | [photosystem II 10 kDa polypeptide, chloroplastic](https://blast.ncbi.nlm.nih.gov/Blast.cgi) | stress response |
| TRIAE_CS42_6AL_TGACv1_473748_AA1532760.1 | 0.510689738 | photosystem II 10 kDa polypeptide, chloroplastic | stress response |
| TRIAE_CS42_6BL_TGACv1_500044_AA1597870.1 | 0.510689738 | photosystem II 10 kDa polypeptide, chloroplastic | stress response |
| TRIAE_CS42_4DL_TGACv1_344347_AA1146290.1 | 0.440867555 | heat shock cognate 70 kDa protein 2-like | stress response |
| TRIAE_CS42_5BS_TGACv1_423362_AA1375070.1 | 0.38082633 | Thaumatin-like protein | stress response |
| TRIAE_CS42_U_TGACv1_643161_AA2128250.1 | 0.38082633 | Thaumatin-like protein | stress response |
| TRIAE_CS42_4AL_TGACv1_290053_AA0980920.1 | 0.371634892 | Ribulose bisphosphate carboxylase/oxygenase activase B, chloroplastic | stress response |
| TRIAE_CS42_4AL_TGACv1_290053_AA0980920.2 | 0.371634892 | Ribulose bisphosphate carboxylase/oxygenase activase B, chloroplastic | stress response |
| TRIAE_CS42_4AL_TGACv1_290053_AA0980920.3 | 0.371634892 | Ribulose bisphosphate carboxylase/oxygenase activase B, chloroplastic | stress response |
| TRIAE_CS42_4AL_TGACv1_290053_AA0980920.4 | 0.371634892 | Ribulose bisphosphate carboxylase/oxygenase activase B, chloroplastic | stress response |
| TRIAE_CS42_4DS_TGACv1_361664_AA1171230.1 | 0.371634892 | Ribulose bisphosphate carboxylase/oxygenase activase B, chloroplastic | stress response |
| TRIAE_CS42_4DS_TGACv1_361664_AA1171230.2 | 0.371634892 | Ribulose bisphosphate carboxylase/oxygenase activase B, chloroplastic | stress response |
| TRIAE_CS42_1BS_TGACv1_052525_AA0181760.1 | 0.340314346 | peroxidase A2-like | redox |
| TRIAE_CS42_4AS_TGACv1_308486_AA1028240.1 | 0.340191694 | vacuolar targeting receptor bp-80 | stress response |
| TRIAE_CS42_4AS_TGACv1_308486_AA1028240.2 | 0.340191694 | vacuolar targeting receptor bp-80 | stress response |
| TRIAE_CS42_4BL_TGACv1_320576_AA1043650.1 | 0.340191694 | vacuolar targeting receptor bp-80 | stress response |
| TRIAE_CS42_4BL_TGACv1_320576_AA1043650.2 | 0.340191694 | vacuolar targeting receptor bp-80 | stress response |
| TRIAE_CS42_U_TGACv1_644195_AA2137980.1 | 0.340191694 | vacuolar-sorting receptor 1-like | stress response |
| TRIAE_CS42_5AL_TGACv1_376019_AA1230850.1 | 0.289136824 | Glycosyltransferase | stress response |
| TRIAE_CS42_5AL_TGACv1_378731_AA1254390.1 | 0.28029847 | mitochondrial outer membrane porin | stress response |
| TRIAE_CS42_5BL_TGACv1_407642_AA1358540.1 | 0.28029847 | mitochondrial outer membrane porin | stress response |
| TRIAE_CS42_4BS_TGACv1_328260_AA1085360.1 | 0.272282483 | Aldehyde dehydrogenase | stress response |
| TRIAE_CS42_4BS_TGACv1_328260_AA1085360.2 | 0.272282483 | Aldehyde dehydrogenase | stress response |
| TRIAE_CS42_4BS_TGACv1_328260_AA1085360.3 | 0.272282483 | Aldehyde dehydrogenase | stress response |
| TRIAE_CS42_4BS_TGACv1_328260_AA1085360.4 | 0.272282483 | Aldehyde dehydrogenase | stress response |
| TRIAE_CS42_3AL_TGACv1_194480_AA0633800.1 | 0.462415059 | uncharacterized protein |  |
| TRIAE_CS42_6AL_TGACv1_474709_AA1535780.1 | 0.297396509 | uncharacterized protein |  |
| Down-regulated | | | |
| AIG90481 | -0.27665402 | NAD(P)H-quinone oxidoreductase subunit I, chloroplastic | plastid |
| TRIAE_CS42_1BL_TGACv1_031408_AA0113420.1 | -0.28273982 | Chlorophyll a-b binding protein, chloroplastic | chloroplast |
| TRIAE_CS42_1DL_TGACv1_062185_AA0210150.1 | -0.28273982 | Chlorophyll a-b binding protein, chloroplastic | chloroplast |
| TRIAE_CS42_2AS_TGACv1_112955_AA0348610.2 | -0.28273982 | Chlorophyll a-b binding protein, chloroplastic | chloroplast |
| TRIAE_CS42_2BS_TGACv1_147491_AA0483780.1 | -0.30618902 | Chlorophyll a-b binding protein, chloroplastic | chloroplast |
| TRIAE_CS42_5AL_TGACv1_374690_AA1206680.1 | -0.58732952 | Chlorophyll a-b binding protein 4, chloroplastic | chloroplast |
| TRIAE_CS42_5AL_TGACv1_374690_AA1206680.2 | -0.58732952 | Chlorophyll a-b binding protein 4, chloroplastic | chloroplast |
| TRIAE_CS42_5AL_TGACv1_374690_AA1206680.5 | -0.58732952 | Chlorophyll a-b binding protein 4, chloroplastic | chloroplast |
| TRIAE_CS42_5BL_TGACv1_404484_AA1301600.1 | -0.58732952 | Chlorophyll a-b binding protein 4, chloroplastic | chloroplast |
| TRIAE_CS42_2AS_TGACv1_112810_AA0345610.1 | -0.68568509 | Chlorophyll a-b binding protein, chloroplastic | chloroplast |
| TRIAE_CS42_2AS_TGACv1_112810_AA0345610.2 | -0.68568509 | Chlorophyll a-b binding protein, chloroplastic | chloroplast |
| TRIAE_CS42_2BS_TGACv1_146599_AA0469070.2 | -0.68568509 | Chlorophyll a-b binding protein, chloroplastic | chloroplast |
| TRIAE_CS42_2BS_TGACv1_146599_AA0469070.3 | -0.68568509 | Chlorophyll a-b binding protein, chloroplastic | chloroplast |
| TRIAE_CS42_2DS_TGACv1_178953_AA0602720.1 | -0.68568509 | Chlorophyll a-b binding protein, chloroplastic | chloroplast |
| TRIAE_CS42_2BL_TGACv1_132610_AA0438610.1 | -1.29582668 | Aminomethyltransferase | redox |
| TRIAE_CS42_6DL_TGACv1_526647_AA1688900.2 | -0.26330284 | uncharacterized protein |  |
| TRIAE_CS42_1AL_TGACv1_000553_AA0014550.1 | -0.54635358 | uncharacterized protein |  |
| TRIAE_CS42_1DL_TGACv1_062818_AA0220440.1 | -0.54635358 | uncharacterized protein |  |
| TRIAE_CS42_6BL_TGACv1_503168_AA1627380.1 | -1.17038162 | uncharacterized protein |  |
| TRIAE_CS42_6BL_TGACv1_503168_AA1627380.2 | -1.17038162 | uncharacterized protein |  |
| TRIAE_CS42_6BL_TGACv1_503168_AA1627380.3 | -1.17038162 | uncharacterized protein |  |
