# Supplementary material for: iTRAQ Proteomic Analysis of Wheat (Triticum aestivum L.) Genotypes Differing in Waterlogging Tolerance
Source: Front Plant Sci. 2022 Apr 25;13:890083. doi: 10.3389/fpls.2022.890083 (PMC9084233; doi:10.3389/fpls.2022.890083)
Supplement: Supplementary file 5 [file Table_2.DOCX]

**TableS2. Differentially expressed proteins in XM55 between WL and CK**

| Gene ID | log2_FC(WL/CK) | Protein Description | Functional Category |
| --- | --- | --- | --- |
| UP-regulated | | | |
| TRIAE_CS42_2AL_TGACv1_093357_AA0278350.1 | 0.766476921 | Calreticulin | stress responses |
| TRIAE_CS42_2AL_TGACv1_093357_AA0278350.2 | 0.766476921 | Calreticulin | stress responses |
| TRIAE_CS42_2AL_TGACv1_093357_AA0278350.3 | 0.766476921 | Calreticulin | stress responses |
| TRIAE_CS42_2BL_TGACv1_130251_AA0407250.1 | 0.766476921 | Calreticulin | stress responses |
| TRIAE_CS42_2BL_TGACv1_130251_AA0407250.2 | 0.766476921 | Calreticulin | stress responses |
| TRIAE_CS42_2BL_TGACv1_130251_AA0407250.3 | 0.766476921 | Calreticulin | stress responses |
| TRIAE_CS42_2DL_TGACv1_159478_AA0538940.1 | 0.766476921 | Calreticulin | stress responses |
| TRIAE_CS42_2DL_TGACv1_159478_AA0538940.3 | 0.766476921 | Calreticulin | stress responses |
| TRIAE_CS42_2DL_TGACv1_159478_AA0538940.4 | 0.766476921 | Calreticulin | stress responses |
| TRIAE_CS42_2DL_TGACv1_159478_AA0538940.5 | 0.766476921 | Calreticulin | stress responses |
| TRIAE_CS42_2BL_TGACv1_130584_AA0414140.1 | 0.386359431 | [ubiquinol oxidase 4, chloroplastic/chromoplastic](https://blast.ncbi.nlm.nih.gov/Blast.cgi) | redox |
| TRIAE_CS42_4BL_TGACv1_320411_AA1038280.1 | 0.332512218 | [probable enoyl-CoA hydratase 1, peroxisomal](https://blast.ncbi.nlm.nih.gov/Blast.cgi) | redox |
| AIG90481 | 0.311569274 | H-quinone oxidoreductase subunit I, chloroplastic | redox |
| TRIAE_CS42_1AL_TGACv1_000099_AA0003250.1 | 0.3039771 | [photosystem I reaction center subunit VI, chloroplastic](https://blast.ncbi.nlm.nih.gov/Blast.cgi) | chloroplast |
| TRIAE_CS42_1BL_TGACv1_030454_AA0091040.1 | 0.3039771 | [photosystem I reaction center subunit VI, chloroplastic](https://blast.ncbi.nlm.nih.gov/Blast.cgi) | chloroplast |
| TRIAE_CS42_1BL_TGACv1_030454_AA0091140.2 | 0.3039771 | [photosystem I reaction center subunit VI, chloroplastic](https://blast.ncbi.nlm.nih.gov/Blast.cgi) | chloroplast |
| TRIAE_CS42_1DL_TGACv1_062668_AA0218050.1 | 0.3039771 | [photosystem I reaction center subunit VI, chloroplastic](https://blast.ncbi.nlm.nih.gov/Blast.cgi) | chloroplast |
| TRIAE_CS42_5AL_TGACv1_376699_AA1239990.1 | 0.303527241 | [preprotein translocase subunit SECY, chloroplastic](https://blast.ncbi.nlm.nih.gov/Blast.cgi) | chloroplast |
| TRIAE_CS42_5AL_TGACv1_376699_AA1239990.2 | 0.303527241 | [preprotein translocase subunit SECY, chloroplastic](https://blast.ncbi.nlm.nih.gov/Blast.cgi) | chloroplast |
| TRIAE_CS42_5AL_TGACv1_376699_AA1239990.3 | 0.303527241 | [preprotein translocase subunit SECY, chloroplastic](https://blast.ncbi.nlm.nih.gov/Blast.cgi) | chloroplast |
| TRIAE_CS42_5DL_TGACv1_432955_AA1395960.1 | 0.303527241 | [preprotein translocase subunit SECY, chloroplastic [Aegilops tauschii subsp. tauschii]](https://blast.ncbi.nlm.nih.gov/Blast.cgi) | chloroplast |
| TRIAE_CS42_5DL_TGACv1_432955_AA1395960.2 | 0.303527241 | [preprotein translocase subunit SECY, chloroplastic](https://blast.ncbi.nlm.nih.gov/Blast.cgi) | chloroplast |
| TRIAE_CS42_4BL_TGACv1_320837_AA1049910.1 | 0.561615189 | [predicted protein](https://blast.ncbi.nlm.nih.gov/Blast.cgi) |  |
| TRIAE_CS42_2AL_TGACv1_094081_AA0292320.2 | 0.46545373 | Uncharacterized protein |  |
| TRIAE_CS42_6AS_TGACv1_485670_AA1549870.1 | 0.388433988 | Uncharacterized protein |  |
| TRIAE_CS42_4BS_TGACv1_328072_AA1082310.1 | 0.377338032 | Uncharacterized protein |  |
| TRIAE_CS42_4DS_TGACv1_361148_AA1161950.1 | 0.377338032 | Uncharacterized protein |  |
| TRIAE_CS42_3AS_TGACv1_211132_AA0685770.1 | 0.363906403 | Uncharacterized protein |  |
| TRIAE_CS42_3B_TGACv1_226896_AA0820020.1 | 0.363906403 | Uncharacterized protein |  |
| TRIAE_CS42_3B_TGACv1_226896_AA0820020.2 | 0.363906403 | Uncharacterized protein |  |
| TRIAE_CS42_3B_TGACv1_226896_AA0820020.3 | 0.363906403 | Uncharacterized protein |  |
| TRIAE_CS42_3B_TGACv1_226896_AA0820020.4 | 0.363906403 | Uncharacterized protein |  |
| Down-regulated | | | |
| TRIAE_CS42_6AL_TGACv1_471755_AA1513800.2 | -0.27007686 | [Protein grpE](https://blast.ncbi.nlm.nih.gov/Blast.cgi) | growth and development |
| TRIAE_CS42_6AS_TGACv1_487122_AA1568340.1 | -0.29214913 | [3-isopropylmalate dehydratase large subunit, chloroplastic](https://blast.ncbi.nlm.nih.gov/Blast.cgi) | chloroplast |
| TRIAE_CS42_4AL_TGACv1_289007_AA0963320.1 | -0.30235134 | [proteasome subunit alpha type-5-like](https://blast.ncbi.nlm.nih.gov/Blast.cgi) | metabolic |
| TRIAE_CS42_4BS_TGACv1_330306_AA1107010.1 | -0.30235134 | [proteasome subunit alpha type-5-like](https://blast.ncbi.nlm.nih.gov/Blast.cgi) | metabolic |
| TRIAE_CS42_4BS_TGACv1_330306_AA1107010.2 | -0.30235134 | [proteasome subunit alpha type-5-like](https://blast.ncbi.nlm.nih.gov/Blast.cgi) | metabolic |
| TRIAE_CS42_1AL_TGACv1_002534_AA0042890.1 | -0.32060795 | solanesyl-diphosphate synthase 2, chloroplastic | chloroplast |
| TRIAE_CS42_1BL_TGACv1_030959_AA0104550.1 | -0.32060795 | [solanesyl-diphosphate synthase 2, chloroplastic](https://blast.ncbi.nlm.nih.gov/Blast.cgi) | chloroplast |
| TRIAE_CS42_1DL_TGACv1_063491_AA0227880.1 | -0.32060795 | [solanesyl-diphosphate synthase 2, chloroplastic](https://blast.ncbi.nlm.nih.gov/Blast.cgi) | chloroplast |
| TRIAE_CS42_1DL_TGACv1_063491_AA0227880.2 | -0.32060795 | [solanesyl-diphosphate synthase 2, chloroplastic](https://blast.ncbi.nlm.nih.gov/Blast.cgi) | chloroplast |
| TRIAE_CS42_7AL_TGACv1_558993_AA1797380.1 | -0.32466768 | [cinnamoyl-CoA reductase 2-like](https://blast.ncbi.nlm.nih.gov/Blast.cgi) | redox |
| TRIAE_CS42_4AL_TGACv1_289784_AA0976760.1 | -0.33114219 | DEAD-box ATP-dependent RNA helicase 3, chloroplastic | metabolic |
| TRIAE_CS42_3AS_TGACv1_211819_AA0694600.3 | -0.38756194 | Hydroxyphenylpyruvate reductase | redox |
| TRIAE_CS42_3DS_TGACv1_272455_AA0920950.1 | -0.38756194 | Hydroxyphenylpyruvate reductase | redox |
| TRIAE_CS42_6AS_TGACv1_485212_AA1540230.1 | -0.40227414 | [peroxidase 70-like](https://blast.ncbi.nlm.nih.gov/Blast.cgi) | redox |
| TRIAE_CS42_2AL_TGACv1_095116_AA0307060.1 | -0.44313582 | probable L-ascorbate peroxidase 7, chloroplastic | redox |
| TRIAE_CS42_6AL_TGACv1_472257_AA1520000.2 | -0.49685378 | Thioredoxin-like protein CDSP32, chloroplastic | metabolic |
| TRIAE_CS42_2AS_TGACv1_113572_AA0358040.1 | -0.57016026 | [peroxidase](https://blast.ncbi.nlm.nih.gov/Blast.cgi) | redox |
| TRIAE_CS42_2AS_TGACv1_113572_AA0358040.2 | -0.57016026 | [peroxidase](https://blast.ncbi.nlm.nih.gov/Blast.cgi) | redox |
| TRIAE_CS42_2AS_TGACv1_114421_AA0367100.1 | -0.57016026 | [peroxidase 3](https://blast.ncbi.nlm.nih.gov/Blast.cgi) | redox |
| TRIAE_CS42_1AS_TGACv1_019295_AA0064480.1 | -0.26303441 | Uncharacterized protein |  |
| TRIAE_CS42_1AS_TGACv1_019295_AA0064480.2 | -0.26303441 | Uncharacterized protein |  |
| TRIAE_CS42_1AS_TGACv1_019295_AA0064480.3 | -0.26303441 | Uncharacterized protein |  |
| TRIAE_CS42_1AS_TGACv1_019295_AA0064480.4 | -0.26303441 | Uncharacterized protein |  |
| TRIAE_CS42_1AS_TGACv1_019295_AA0064480.5 | -0.26303441 | Uncharacterized protein |  |
| TRIAE_CS42_1AS_TGACv1_019295_AA0064480.6 | -0.26303441 | Uncharacterized protein |  |
| TRIAE_CS42_1AS_TGACv1_019295_AA0064480.7 | -0.26303441 | Uncharacterized protein |  |
| TRIAE_CS42_1AS_TGACv1_019295_AA0064480.8 | -0.26303441 | Uncharacterized protein |  |
| TRIAE_CS42_1DS_TGACv1_081075_AA0257430.1 | -0.26303441 | Uncharacterized protein |  |
| TRIAE_CS42_1DS_TGACv1_081075_AA0257430.2 | -0.26303441 | Uncharacterized protein |  |
| TRIAE_CS42_1DS_TGACv1_081075_AA0257430.3 | -0.26303441 | Uncharacterized protein |  |
| TRIAE_CS42_1DS_TGACv1_081075_AA0257430.4 | -0.26303441 | Uncharacterized protein |  |
| TRIAE_CS42_1DS_TGACv1_081075_AA0257430.5 | -0.26303441 | Uncharacterized protein |  |
| TRIAE_CS42_1DS_TGACv1_081075_AA0257430.6 | -0.26303441 | Uncharacterized protein |  |
| TRIAE_CS42_3AS_TGACv1_211670_AA0692840.1 | -0.26333913 | [unnamed protein product](https://blast.ncbi.nlm.nih.gov/Blast.cgi) |  |
| TRIAE_CS42_7AS_TGACv1_571064_AA1844100.1 | -0.26475088 | Uncharacterized protein |  |
| TRIAE_CS42_7BS_TGACv1_593340_AA1950600.1 | -0.26475088 | Uncharacterized protein |  |
| TRIAE_CS42_2DS_TGACv1_177526_AA0579360.1 | -0.29133095 | [unnamed protein product](https://blast.ncbi.nlm.nih.gov/Blast.cgi) |  |
| TRIAE_CS42_2DS_TGACv1_177526_AA0579360.2 | -0.29133095 | [unnamed protein product](https://blast.ncbi.nlm.nih.gov/Blast.cgi) |  |
| TRIAE_CS42_2DS_TGACv1_177526_AA0579360.3 | -0.29133095 | [unnamed protein product](https://blast.ncbi.nlm.nih.gov/Blast.cgi) |  |
| TRIAE_CS42_6DL_TGACv1_527764_AA1708330.1 | -0.30116239 | Uncharacterized protein |  |
| TRIAE_CS42_2AS_TGACv1_113542_AA0357510.1 | -0.35069207 | Uncharacterized protein |  |
| TRIAE_CS42_2BS_TGACv1_148446_AA0492900.1 | -0.35069207 | Uncharacterized protein |  |
| TRIAE_CS42_2DS_TGACv1_178892_AA0602250.1 | -0.35069207 | Uncharacterized protein |  |
| TRIAE_CS42_1BL_TGACv1_032389_AA0129290.1 | -0.35508519 | Uncharacterized protein |  |
| TRIAE_CS42_2AS_TGACv1_112463_AA0338530.1 | -0.36597405 | Uncharacterized protein |  |
| TRIAE_CS42_U_TGACv1_641432_AA2094970.1 | -0.38432288 | unkown |  |
| TRIAE_CS42_5BS_TGACv1_423595_AA1380330.1 | -0.39055364 | Uncharacterized protein |  |
| TRIAE_CS42_5BS_TGACv1_423595_AA1380330.3 | -0.39055364 | Uncharacterized protein |  |
| TRIAE_CS42_4DL_TGACv1_342489_AA1115100.5 | -0.44057259 | Uncharacterized protein |  |
