# Supplementary material for: iTRAQ Proteomic Analysis of Wheat (Triticum aestivum L.) Genotypes Differing in Waterlogging Tolerance
Source: Front Plant Sci. 2022 Apr 25;13:890083. doi: 10.3389/fpls.2022.890083 (PMC9084233; doi:10.3389/fpls.2022.890083)
Supplement: Supplementary file 6 [file Table_3.DOCX]

**TableS3. Differentially expressed proteins in YM158 between WL and CK**

| Gene ID | log2_FC(WL/CK) | Protein Description | | Functional Category |  |  |
| --- | --- | --- | --- | --- | --- | --- |
| UP-regulated | | | | |  |  |
| TRIAE_CS42_2BL_TGACv1_131998_AA0434300.1 | 0.509707606 | Carboxypeptidase | | metabolic |  |  |
| TRIAE_CS42_5AL_TGACv1_376019_AA1230850.1 | 0.280552167 | Glycosyltransferase | | metabolic |  |  |
| TRIAE_CS42_7AS_TGACv1_569693_AA1821940.1 | 0.352030127 | hypothetical protein | |  |  |  |
| TRIAE_CS42_4BL_TGACv1_320837_AA1049910.1 | 0.490230478 | predicted protein | |  |  |  |
| TRIAE_CS42_2AL_TGACv1_094081_AA0292320.2 | 0.4423414 | Uncharacterized protein | |  |  |  |
| TRIAE_CS42_4BS_TGACv1_329474_AA1101780.1 | 0.435063523 | [predicted protein](https://blast.ncbi.nlm.nih.gov/Blast.cgi) | |  |  |  |
| TRIAE_CS42_4BS_TGACv1_329474_AA1101780.3 | 0.435063523 | [predicted protein](https://blast.ncbi.nlm.nih.gov/Blast.cgi) | |  |  |  |
| TRIAE_CS42_1AL_TGACv1_002075_AA0038500.1 | 0.40152957 | Uncharacterized protein | |  |  |  |
| TRIAE_CS42_1AL_TGACv1_002075_AA0038500.2 | 0.40152957 | Uncharacterized protein | |  |  |  |
| TRIAE_CS42_6AS_TGACv1_485670_AA1549870.1 | 0.314682113 | Uncharacterized protein | |  |  |  |
| TRIAE_CS42_4AL_TGACv1_288845_AA0959550.1 | 0.285455908 | Uncharacterized protein | |  |  |  |
| TRIAE_CS42_5BL_TGACv1_405828_AA1335990.1 | 0.285455908 | Uncharacterized protein | |  |  |  |
| TRIAE_CS42_5DL_TGACv1_435646_AA1453010.1 | 0.285455908 | Uncharacterized protein | |  |  |  |
| Down-regulated | | | | |  |  |
| TRIAE_CS42_4BS_TGACv1_328833_AA1094200.2 | -0.263525202 | | Aminopeptidase | catabolic |  |  |
| TRIAE_CS42_4BS_TGACv1_328833_AA1094200.3 | -0.263525202 | | Aminopeptidase | catabolic |  |  |
| TRIAE_CS42_1AL_TGACv1_002534_AA0042890.1 | -0.267560436 | | solanesyl-diphosphate synthase 2, chloroplastic | chloroplast |  |  |
| TRIAE_CS42_1BL_TGACv1_030959_AA0104550.1 | -0.267560436 | | [solanesyl-diphosphate synthase 2, chloroplastic](https://blast.ncbi.nlm.nih.gov/Blast.cgi) | chloroplast |  |  |
| TRIAE_CS42_1DL_TGACv1_063491_AA0227880.1 | -0.267560436 | | [solanesyl-diphosphate synthase 2, chloroplastic](https://blast.ncbi.nlm.nih.gov/Blast.cgi) | chloroplast |  |  |
| TRIAE_CS42_1DL_TGACv1_063491_AA0227880.2 | -0.267560436 | | [solanesyl-diphosphate synthase 2, chloroplastic](https://blast.ncbi.nlm.nih.gov/Blast.cgi) | chloroplast |  |  |
| TRIAE_CS42_3B_TGACv1_221690_AA0747520.1 | -0.274196878 | | PsbP domain-containing protein 6, chloroplastic | metabolic |  |  |
| TRIAE_CS42_3DL_TGACv1_250418_AA0867860.1 | -0.274196878 | | PsbP domain-containing protein 6, chloroplastic | metabolic |  |  |
| TRIAE_CS42_4AL_TGACv1_289784_AA0976760.1 | -0.283679679 | | DEAD-box ATP-dependent RNA helicase 3, chloroplastic | metabolic |  |  |
| TRIAE_CS42_7AL_TGACv1_558524_AA1794020.1 | -0.288476783 | | DAG protein, chloroplastic-like | chloroplast |  |  |
| TRIAE_CS42_7AL_TGACv1_558524_AA1794020.2 | -0.288476783 | | DAG protein, chloroplastic-like | chloroplast |  |  |
| TRIAE_CS42_7AL_TGACv1_558524_AA1794020.3 | -0.288476783 | | DAG protein, chloroplastic-like | chloroplast |  |  |
| TRIAE_CS42_7BL_TGACv1_577507_AA1877300.1 | -0.288476783 | | [multiple organellar RNA editing factor 9, chloroplastic](https://blast.ncbi.nlm.nih.gov/Blast.cgi) | chloroplast |  |  |
| TRIAE_CS42_7BL_TGACv1_577507_AA1877300.2 | -0.288476783 | | [multiple organellar RNA editing factor 9, chloroplastic](https://blast.ncbi.nlm.nih.gov/Blast.cgi) | chloroplast |  |  |
| TRIAE_CS42_7BL_TGACv1_577507_AA1877300.3 | -0.288476783 | | [multiple organellar RNA editing factor 9, chloroplastic](https://blast.ncbi.nlm.nih.gov/Blast.cgi) | chloroplast |  |  |
| TRIAE_CS42_7DL_TGACv1_602578_AA1961500.1 | -0.288476783 | | [multiple organellar RNA editing factor 9, chloroplastic](https://blast.ncbi.nlm.nih.gov/Blast.cgi) | chloroplast |  |  |
| TRIAE_CS42_7DL_TGACv1_602578_AA1961500.2 | -0.288476783 | | [multiple organellar RNA editing factor 9, chloroplastic](https://blast.ncbi.nlm.nih.gov/Blast.cgi) | chloroplast |  |  |
| TRIAE_CS42_7DL_TGACv1_602578_AA1961500.3 | -0.288476783 | | [multiple organellar RNA editing factor 9, chloroplastic](https://blast.ncbi.nlm.nih.gov/Blast.cgi) | chloroplast |  |  |
| TRIAE_CS42_7DL_TGACv1_602578_AA1961500.4 | -0.288476783 | | [multiple organellar RNA editing factor 9, chloroplastic](https://blast.ncbi.nlm.nih.gov/Blast.cgi) | chloroplast |  |  |
| TRIAE_CS42_3AL_TGACv1_195570_AA0651350.1 | -0.31447229 | | Fe-S cluster assembly factor HCF101, chloroplastic | chloroplast |  |  |
| TRIAE_CS42_3AL_TGACv1_195570_AA0651350.2 | -0.31447229 | | Fe-S cluster assembly factor HCF101, chloroplastic | chloroplast |  |  |
| TRIAE_CS42_3AL_TGACv1_195570_AA0651350.3 | -0.31447229 | | Fe-S cluster assembly factor HCF101, chloroplastic | chloroplast |  |  |
| TRIAE_CS42_3DL_TGACv1_250912_AA0874940.1 | -0.31447229 | | [fe-S cluster assembly factor HCF101, chloroplastic isoform X1](https://blast.ncbi.nlm.nih.gov/Blast.cgi) | chloroplast |  |  |
| TRIAE_CS42_3DL_TGACv1_250912_AA0874940.2 | -0.31447229 | | [fe-S cluster assembly factor HCF101, chloroplastic isoform X1](https://blast.ncbi.nlm.nih.gov/Blast.cgi) | chloroplast |  |  |
| TRIAE_CS42_5AL_TGACv1_374690_AA1206680.1 | -0.314957245 | | Chlorophyll a-b binding protein 4, chloroplastic | stress response |  |  |
| TRIAE_CS42_5AL_TGACv1_374690_AA1206680.2 | -0.314957245 | | Chlorophyll a-b binding protein 4, chloroplastic | stress response |  |  |
| TRIAE_CS42_5AL_TGACv1_374690_AA1206680.5 | -0.314957245 | | Chlorophyll a-b binding protein 4, chloroplastic | stress response |  |  |
| TRIAE_CS42_5BL_TGACv1_404484_AA1301600.1 | -0.314957245 | | Chlorophyll a-b binding protein 4, chloroplastic | stress response |  |  |
| TRIAE_CS42_4AL_TGACv1_291921_AA0997070.1 | -0.328991487 | | acyl-CoA-binding protein-like | stress response |  |  |
| TRIAE_CS42_2BL_TGACv1_130632_AA0415000.1 | -0.355889613 | | thylakoid lumenal 29 kDa protein, chloroplastic | stress response |  |  |
| TRIAE_CS42_6AS_TGACv1_487122_AA1568340.1 | -0.418426951 | | [3-isopropylmalate dehydratase large subunit, chloroplastic](https://blast.ncbi.nlm.nih.gov/Blast.cgi) | chloroplast |  |  |
| TRIAE_CS42_7BL_TGACv1_578978_AA1902600.1 | -0.482933438 | | [dolichyl-diphosphooligosaccharide--protein glycosyltransferase 48 kDa subunit](https://blast.ncbi.nlm.nih.gov/Blast.cgi) | metabolic |  |  |
| TRIAE_CS42_7BL_TGACv1_578978_AA1902600.3 | -0.482933438 | | [dolichyl-diphosphooligosaccharide--protein glycosyltransferase 48 kDa subunit](https://blast.ncbi.nlm.nih.gov/Blast.cgi) | metabolic |  |  |
| TRIAE_CS42_7DL_TGACv1_605357_AA2006220.1 | -0.482933438 | | [dolichyl-diphosphooligosaccharide--protein glycosyltransferase 48 kDa subunit](https://blast.ncbi.nlm.nih.gov/Blast.cgi) | metabolic |  |  |
| TRIAE_CS42_2BL_TGACv1_131439_AA0427700.2 | -0.488747185 | | Superoxide dismutase [Mn], mitochondrial | redox |  |  |
| TRIAE_CS42_2AL_TGACv1_093548_AA0282400.1 | -0.589265845 | | Aminomethyltransferase, mitochondrial | redox |  |  |
| TRIAE_CS42_2DS_TGACv1_177289_AA0572380.1 | -0.276311518 | | [predicted protein](https://blast.ncbi.nlm.nih.gov/Blast.cgi) |  |  |  |
| TRIAE_CS42_2DS_TGACv1_177289_AA0572380.2 | -0.276311518 | | [predicted protein](https://blast.ncbi.nlm.nih.gov/Blast.cgi) |  |  |  |
| TRIAE_CS42_4AS_TGACv1_306673_AA1011900.1 | -0.289333751 | | uncharacterized protein |  |  |  |
| TRIAE_CS42_4DL_TGACv1_343777_AA1139610.1 | -0.289333751 | | uncharacterized protein |  |  |  |
| TRIAE_CS42_6BS_TGACv1_514577_AA1661740.1 | -0.316296227 | | Uncharacterized protein |  |  |  |
| TRIAE_CS42_7DL_TGACv1_606336_AA2009860.1 | -0.324626586 | | Uncharacterized protein |  |  |  |
| TRIAE_CS42_7DL_TGACv1_606336_AA2009860.2 | -0.324626586 | | Uncharacterized protein |  |  |  |
| TRIAE_CS42_3B_TGACv1_222346_AA0762680.1 | -0.34684867 | | Uncharacterized protein |  |  |  |
| TRIAE_CS42_3DL_TGACv1_249129_AA0838870.1 | -0.34684867 | | Uncharacterized protein |  |  |  |
| TRIAE_CS42_3DL_TGACv1_249129_AA0838870.2 | -0.34684867 | | Uncharacterized protein |  |  |  |
