# Supplementary material for: iTRAQ Proteomic Analysis of Wheat (Triticum aestivum L.) Genotypes Differing in Waterlogging Tolerance
Source: Front Plant Sci. 2022 Apr 25;13:890083. doi: 10.3389/fpls.2022.890083 (PMC9084233; doi:10.3389/fpls.2022.890083)
Supplement: Supplementary file 9 [file Table_6.DOCX]

**TableS6. qRT-PCR primers for analysis of mRNA expression**

| Target | GeneID | Primer orientation | Primer sequence |
| --- | --- | --- | --- |
| O1/2 | TRIAE_CS42_2BL_TGACv1_130584_AA0414140.1 | Forward | GGGACTACGCCAGGTTCTTC |
|  |  | Reverse | AGCCATACAGAGTTGCCACC |
| O3/4 | TRIAE_CS42_2BL_TGACv1_131039_AA0421600.2 | Forward | ACCATCACCAACGACAAGGG |
|  |  | Reverse | GATGGTGTTGCGCATGTTGT |
| O5/6 | TRIAE_CS42_2BL_TGACv1_131439_AA0427700.2 | Forward | CGCCACCTACGTCGCCAACT |
|  |  | Reverse | GTGCCTCACCACCACCCTCG |
| O7/8 | TRIAE_CS42_2BL_TGACv1_132610_AA0438610.1 | Forward | TGGAGCAGCACATCACGCCA |
|  |  | Reverse | CGCTCGTCACCTCCCCGATG |
| O9/10 | TRIAE_CS42_3AL_TGACv1_195570_AA0651350.1 | Forward | CGCCCGCTCCTCATCTCGTC |
|  |  | Reverse | TGAGCACGTCCCTCTTGGCA |
| O11/12 | TRIAE_CS42_3AS_TGACv1_211332_AA0688720.1 | Forward | TCAAGGACGAGCGGCTGGTG |
|  |  | Reverse | TCCTTGTCAGCAGCGTCCACC |
| O13/14 | TRIAE_CS42_4BL_TGACv1_321826_AA1065960.1 | Forward | AGGACGCGCACATCTCCGAC |
|  |  | Reverse | GCCAGCCACGTCGACCAGAT |
| O15/16 | TRIAE_CS42_4BS_TGACv1_329474_AA1101780.1 | Forward | TTCGTGCAGACCAGGCTCCG |
|  |  | Reverse | GCTTGGGGTGGACGGAGCTT |
| O17/18 | TRIAE_CS42_4BS_TGACv1_330468_AA1107820.2 | Forward | GAGGGAGCCGGCGAATGGAT |
|  |  | Reverse | GCGTCTTAGCGGTCGGGGAA |
| O19/20 | TRIAE_CS42_5DS_TGACv1_456540_AA1473460.1 | Forward | ACCAACCCACCCACACCGAC |
|  |  | Reverse | GCGAAGTTGCCGGAGAGCCA |
| O21/22 | TRIAE_CS42_6BL_TGACv1_503168_AA1627380.1 | Forward | TGGCCAGCATCGACCTCACC |
|  |  | Reverse | GAAGGAGGAGGAGGCGTCGC |
| O23/24 | TRIAE_CS42_1AL_TGACv1_000099_AA0003250.1 | Forward | CAAGTACGGGGAGAAGAGCG |
|  |  | Reverse | CCTTCTTGATGGGGAGCAGG |
| O25/26 | TRIAE_CS42_2AL_TGACv1_093357_AA0278350.1 | Forward | CAGAGTACAAGGGCCCATGG |
|  |  | Reverse | CTTGGCCCAGGTCTCTTCAG |
| O27/28 | TRIAE_CS42_2AL_TGACv1_095116_AA0307060.1 | Forward | CATTGGATGATTAGCGCCGC |
|  |  | Reverse | GTCATCGGAGCAGGTTCACA |
| O29/30 | TRIAE_CS42_2AS_TGACv1_113572_AA0358040.1 | Forward | CGGACCAGGTGCTCTTCAAT |
|  |  | Reverse | CACGAGTTCACCTTGGAGCA |
| O31/32 | TRIAE_CS42_2BL_TGACv1_130251_AA0407250.1 | Forward | CAGAGTACAAGGGCCCATGG |
|  |  | Reverse | CCCAGGTCTCTTCGGCAAAT |
| O33/34 | TRIAE_CS42_2BL_TGACv1_130584_AA0414140.1 | Forward | GGGACTACGCCAGGTTCTTC |
|  |  | Reverse | AGCCATACAGAGTTGCCACC |
| O35/36 | TRIAE_CS42_3AS_TGACv1_211819_AA0694600.3 | Forward | AGGGCCTGCTCATCAACATC |
|  |  | Reverse | ACACACCACGCCCTATCATG |
| O37/38 | TRIAE_CS42_6AS_TGACv1_485212_AA1540230.1 | Forward | CTGTGACGGGTCCATTCTCC |
|  |  | Reverse | GAGCAGCTCCACGTTCTTCT |
| O39/40 | TRIAE_CS42_2AL_TGACv1_093548_AA0282400.1 | Forward | TCTTGCTGCACCAACTCTCC |
|  |  | Reverse | CACCTTGCCCTCGGATTTCT |
| O41/42 | TRIAE_CS42_2BL_TGACv1_131439_AA0427700.2 | Forward | GGGTGTGGCTAGCTTTGGAT |
|  |  | Reverse | CGGCCTCACGTTCTTGTACT |
| O43/44 | TRIAE_CS42_3DL_TGACv1_250912_AA0874940.1 | Forward | GACATCGTCTCGTGTGGGTT |
|  |  | Reverse | CATGGAAGTGCTGCAACGAC |
| O45/46 | TRIAE_CS42_4AL_TGACv1_291921_AA0997070.1 | Forward | GCTGAAATTTCTCTGGCGGC |
|  |  | Reverse | AGAGGACAAGGAATTCGCCG |
| O47/48 | TRIAE_CS42_5AL_TGACv1_374690_AA1206680.1 | Forward | CGGTGGCAGGACATCAAGAA |
|  |  | Reverse | GCGAGCTCCTTCTCCTTGTT |
| O19/50 | TRIAE_CS42_6AS_TGACv1_487122_AA1568340.1 | Forward | CCTCAGCGACTTCAGAGCAA |
|  |  | Reverse | CCTGCATCAGTGTTCCCGAT |
| O51/52 | TRIAE_CS42_7AL_TGACv1_558524_AA1794020.1 | Forward | CGACACCTACCTCAACACCC |
|  |  | Reverse | CCTCCGCTCCTTTGGTTGAT |
| *O53/54* | TRIAE_CS42_7BL_TGACv1_577507_AA1877300.1 | Forward | CGACACCTACCTCAACACCC |
|  |  | Reverse | CCTCCGCTCCTTTGGTTGAT |
| *O55/56* | TRIAE_CS42_7DL_TGACv1_605357_AA2006220.1 | Forward | GGACCCGGAAGCAATGGTTA |
|  |  | Reverse | TGGAACGGATGCCAGCTTAG |
